# Dysfunctional default mode and visual networks underlie cognitive deficits in dementia with Lewy bodies: a resting-state fMRI study

**DOI:** 10.3389/fnagi.2025.1630826

**Published:** 2025-11-19

**Authors:** Zhou Su, Mengran Liu, Jun Kuai, Tingting Yi, Yuechang Zheng, Xinran Bao, Jiyu Ji

**Affiliations:** 1 Department of Neurology, The First Affiliated Hospital of Xinxiang Medical University, Xinxiang, Henan, China; 2 School of Education, University of Bristol, Bristol, United Kingdom; 3 Department of Gastroenterology, The First Affiliated Hospital of Xinxiang Medical University, Xinxiang, Henan, China; 4 Department of Neurology, First Hospital of Qinhuangdao, Hebei, China

**Keywords:** resting-state fMRI, dementia with Lewy bodies, functional connectivity, cognitive impairment, default mode network, graph theory

## Abstract

**Objective:**

To characterize abnormal functional connectivity in dementia with Lewy bodies (DLB) and its association with cognitive impairment using resting-state functional magnetic resonance imaging (rs-fMRI).

**Methods:**

Sixty-eight DLB patients and 38 age-, sex-, and education-matched healthy controls underwent neuropsychological assessments (MoCA, MMSE) and rs-fMRI. Imaging analyses included seed-based functional connectivity (sFC), independent component analysis (ICA), regional homogeneity (ReHo), fractional amplitude of low-frequency fluctuations (fALFF), and graph-theoretical network metrics (small-worldness, global/local efficiency).

**Results:**

DLB patients exhibited significantly reduced FC in the default mode network (DMN) and visual network, including PCC–AG (*P* < 0.001) and PCC–mPFC (*P* < 0.001). ReHo and fALFF indicated decreased local neural synchronization and low-frequency activity in the posterior occipital lobe (fALFF: *P* = 0.004), angular gyrus (fALFF: *P* = 0.001), left temporal pole (fALFF: *P* < 0.001), left parietal (ReHo: *P* < 0.001), and posterior cerebellar lobe (ReHo: *P* < 0.001). Graph theory revealed impaired global network topology in DLB, with decreased small-worldness (*P* < 0.001) and global efficiency (*P* < 0.001). PCC–AG connectivity positively correlated with the MoCA total score (*r* = 0.53, *P* < 0.001), attention (*r* = 0.46, *P* < 0.001), executive (*r* = 0.41, *P* < 0.001), and language function (*r* = 0.34, *P* < 0.001). Posterior occipital fALFF and left parietal ReHo showed significant positive correlations with multiple cognitive domains, including visuospatial ability (*r* = 0.34, *P* < 0.001 for fALFF; *r* = 0.42, *P* < 0.001 for ReHo) and memory (*r* = 0.45, *P* < 0.001 for fALFF; *r* = 0.27, *P* = 0.006 for ReHo). A combined model of PCC–AG connectivity, fALFF, and small-worldness predicted 42% of MoCA variance (*R*^2^ = 0.42, *P* < 0.001).

**Conclusion:**

DLB is characterized by DMN and visual network dysfunction, disrupted local neural activity, and impaired global network integration. These rs-fMRI metrics may serve as potential biomarkers for cognitive deficits in DLB.

## 1 Introduction

Dementia with Lewy bodies (DLB), the second most prevalent neurodegenerative dementia after Alzheimer’s disease (AD) (Vann Jones and O’Brien, 2014), is characterized by heterogeneous clinical features encompassing cognitive decline, motor disturbances, and neuropsychiatric symptoms (McKeith et al., 2017). Despite its distinct clinical profile but with comorbid symptoms, DLB could be misdiagnosed as AD (Bousiges and Blanc, 2022; Yamada et al., 2022). In contrast, misdiagnosis between DLB and Parkinson’s disease dementia (PDD) is less common in clinical practice since their differentiation relies on the clear “1-year rule” regarding the onset sequence of motor vs. cognitive symptoms. Nevertheless, ongoing debate exists about whether DLB and PDD represent distinct entities within Lewy body disorders, given shared α-synuclein pathology (Walker et al., 2015; Fu and Halliday, 2025). Elucidating the neural mechanisms underlying cognitive impairment in DLB and identifying reliable neuroimaging biomarkers are therefore critical for improving clinical outcomes.

Resting-state functional magnetic resonance imaging (rs-fMRI), a non-invasive and reproducible neuroimaging technique (Lowther et al., 2014), has emerged as a powerful tool for investigating brain functional connectivity (FC) by capturing spontaneous low-frequency fluctuations in blood oxygen level-dependent signals (Geng et al., 2024; Cieri et al., 2024). Previous rs-fMRI studies have revealed disease-specific network alterations: AD is associated with FC disruptions in the default mode network (DMN), executive control network, and limbic system (Grieder et al., 2018; Yu et al., 2017). Similarly, PDD and DLB exhibit DMN dysfunction, with additional involvement of the basal ganglia network, reflecting shared and distinct network alterations compared to AD (Piramide et al., 2024; Caminiti et al., 2024). However, systematic investigations into whole-brain network abnormalities in DLB remain limited, particularly those integrating multimodal connectivity metrics, such as seed-based FC, regional homogeneity (ReHo), and fractional amplitude of low-frequency fluctuations (fALFF) with graph-theoretical analysis to dissect domain-specific cognitive deficits (Peraza et al., 2014; Matar et al., 2022).

Cognitive impairment in DLB manifests as multidimensional dysfunction, prominently affecting attention, executive function, and visuospatial abilities, which rely on the coordinated integration of distributed functional networks (McKeith et al., 2017; Schumacher et al., 2019). To address this gap, we conducted a comprehensive rs-fMRI study combining whole-brain FC analysis and graph theory to quantify topological network properties (e.g., small-worldness, global/local efficiency). Our objectives were threefold: (1) to characterize key patterns of disrupted functional connectivity in DLB, (2) to explore associations between these imaging markers and multidimensional cognitive deficits, and (3) to unravel the neurophysiological mechanisms underlying network-level dysfunction. This multimodal approach aims to provide novel insights into early diagnosis and cognitive evaluation in DLB, offering a robust framework for future biomarker development.

## 2 Materials and methods

### 2.1 Subject characteristics

This cross-sectional observational study enrolled 68 patients with DLB diagnosed at the Department of Neurology outpatient and inpatient units of our hospital between January 2021 and December 2024. A control group of 38 age-, sex-, and education-matched healthy older adults was recruited during the same period. For DLB patients, age at assessment was defined as the time of initial diagnosis; for controls, as the date of study enrollment. DLB patients were diagnosed according to the fourth criteria for the diagnosis and management of dementia with Lewy bodies (McKeith et al., 2017). These patients were initially present with at least two core clinical features of DLB (fluctuating cognition, visual hallucinations, parkinsonism, and/or rapid eye movement sleep behavior disorder) or one core clinical feature with at least one indicative biomarker including reduced dopamine transporter uptake in the basal ganglia demonstrated by single-photon emission computed tomography (SPECT) or positron emission tomography-computed tomography (PET), abnormal (low uptake) 123-Iodine-MIBG myocardial scintigraphy, and RBD screening questionnaire (RBD-SQ) and/or polysomnographic confirmation of RBD. The enrolled patients showed relative preservation of medial temporal lobe structures on MRI. All clinical diagnoses of dementia were confirmed by consensus agreement of at least two experienced neurologists, following a case review according to the protocol. Patients meeting any of the following exclusion criteria were excluded from this study: (1) Presence of severe neurological or psychiatric conditions that would impede compliance with study protocols, including severe visual/auditory impairment, aphasia, limb paralysis, or severe mental disorders; (2) Inability to complete required clinical evaluations including neuropsychological assessments, neuroimaging examinations, polysomnography, or other procedures due to aforementioned conditions; (3) Lack of reliable caregivers to provide necessary clinical information or assist with study participation; (4) Patients with acute cardiovascular or cerebrovascular events (e.g., (a) myocardial infarction; (b) disabling stroke (mRS ≥ 3); (c) acute stroke within 6 months); (5) Those diagnosed with neurodegenerative disorders potentially associated with dementia, including PD, AD, Frontotemporal dementia, Multiple system atrophy, Progressive supranuclear palsy, or Corticobasal degeneration. Informed consent was obtained from all participants or their legal guardians in accordance with the ethical principles of the Helsinki Declaration.

This present study collected demographic data, clinical symptoms. Cognitive function was assessed by trained neuropsychological assessors using the Montreal Cognitive Assessment (MoCA) (Nasreddine et al., 2005) and Mini-Mental State Examination (MMSE) (Folstein et al., 1975).

### 2.2 Image data acquisition

All participants underwent rs-fMRI and high-resolution T1-weighted structural imaging using a Siemens 3.0T MRI scanner (Erlangen, Germany). The rs-fMRI data were acquired using an echo-planar imaging (EPI) sequence with the following parameters: repetition time (TR) = 2,000 ms, echo time (TE) = 30 ms, flip angle = 90°, field of view (FOV) = 240 × 240 mm^2^, matrix size = 64 × 64, slice thickness = 4 mm, no inter-slice gap, and 240 volumes. High-resolution T1-weighted images were obtained with a 3D magnetization-prepared rapid gradient-echo sequence: TR = 1,900 ms, TE = 2.52 ms, 176 sagittal slices, and isotropic voxel size = 1 mm^3^. Participants were instructed to remain awake with eyes closed during scanning. Data were reacquired if motion artifacts or drowsiness (monitored via post-scan questionnaires) were detected.

### 2.3 Data preprocessing

Functional imaging data were preprocessed using the DPABI v5.1 toolkit^1^ on MATLAB R2018a (MathWorks, Natick, MA, United States). The standardized pipeline included the following steps: First, the initial 10 volumes were discarded to eliminate transient signal instability during scanner equilibrium. Subsequently, temporal correction (slice timing adjustment) and spatial correction (rigid-body head motion realignment) were performed. The functional images were then co-registered to individual T1-weighted structural images and spatially normalized to the Montreal Neurological Institute 152 template using non-linear transformation (resampled to 3 mm^3^ isotropic voxels). Spatial smoothing was applied with a Gaussian kernel [full width at half maximum (FWHM) = 6 mm], followed by linear detrending and bandpass filtering (0.01–0.08 Hz) to retain low-frequency oscillations. Nuisance covariates, including 24 head motion parameters (Friston model), cerebrospinal fluid, white matter signals, and global mean signal, were regressed out. To control for motion artifacts, framewise displacement (FD) was calculated, and datasets with > 20% of volumes exceeding an FD threshold of 0.2 mm were excluded.

### 2.4 Functional connectivity and network analysis

This study employed multiple analytical methods to evaluate brain functional connectivity and local activity levels, following established protocols for multiparametric imaging in neurodegenerative diseases (Zhou, 2019). Firstly, seed-based functional connectivity (sFC) analysis was conducted using a region-to-region correlation approach. Mean timecourses were extracted from DMN seed regions [PCC: MNI (−5, −49, 40); AG: (−45, −67, 36)] and correlated with timecourses of other regions (e.g., mPFC) using Pearson correlation, followed by Fisher’s Z-transformation. Pearson correlation coefficients for region-to-region connectivity were Fisher’s Z-transformed to ensure normal distribution for subsequent statistical analyses. Seed-based FC analysis targeted connectivity from PCC and AG to other DMN regions [mPFC: MNI (0, 54, 18); contralateral AG; inferior parietal lobule] and visual network regions (posterior occipital cortex), defined using the AAL atlas. Multiple comparisons were corrected using FDR (*P* < 0.05). Secondly, independent component analysis (ICA) was conducted using the Group ICA of fMRI Toolbox (GIFT v4.0b),^2^ decomposing the data into 10–20 intrinsic connectivity networks (e.g., DMN, visual network, executive control network), with between-group comparisons of spatial Z-score distributions. Thirdly, ReHo and fALFF were calculated to quantify local neural synchronization and low-frequency oscillation amplitudes. Finally, graph-theoretical analysis was implemented via GRETNA v2.0.0.^3^ Whole-brain functional connectivity matrices were constructed based on the Automated Anatomical Labeling (AAL-116) atlas. Connections were defined using weighted correlation matrices, retaining connection strength without thresholding, to compute topological parameters including small-worldness coefficient (σ), global efficiency (Eglob), local efficiency (Eloc), average path length (L), and modularity (Q). Additionally, GICA was performed with 15 components (range 10–20) using spatial ICA, followed by back-reconstruction to individual subject Z-maps, with group differences assessed via two-sample *t*-tests and FDR correction.

### 2.5 Group independent component analysis

GICA was performed using the GIFT toolbox (v 4.0b) following established protocols. Data Reduction: Two-stage principal component analysis (PCA) retaining 100 individual-level components and 20 group-level components (determined via minimum description length criteria); Algorithm: Infomax algorithm with 20 ICASSO iterations for stability (clustering similarity threshold > 0.8); Data Reduction: Two-stage principal component analysis (PCA) retaining 100 individual-level components and 20 group-level components (determined via minimum description length criteria); Algorithm: Infomax algorithm with 20 ICASSO iterations for stability (clustering similarity threshold > 0.8); Component Selection: Identification of resting-state networks (e.g., default mode network [DMN], visual, executive control, and salience networks) via spatial template matching (*r* > 0.4).

### 2.6 Statistical analyses

All statistical analyses and data management were performed using SPSS 26.0 for Mac (IBM Corporation, Armonk, NY, United States). Continuous variables were expressed as mean ± standard deviation (SD) when normally distributed or as median (interquartile range) for non-normally distributed data. Between-group comparisons were performed using Student’s *t*-test for parametric data and the Mann-Whitney U test for non-parametric data. Categorical variables were summarized as frequencies (n) with percentages (%) and analyzed using χ^2^ test as appropriate. Ordinal data were presented as median (quartiles) and analyzed with the Mann-Whitney U test. Spearman correlation analysis was performed to examine the relationships between functional connectivity strength, fALFF, ReHo, and both the total MoCA score and its domain-specific cognitive subscales. Subsequently, multiple linear regression model was constructed with the total MoCA score as the dependent variable, incorporating multiple neuroimaging parameters (e.g., PCC-AG connectivity strength, posterior occipital fALFF, and small-worldness coefficient) as independent variables and adjusting for potential confounders including age, sex, and education years. Multiple comparisons in functional connectivity analyses were corrected using the false discovery rate (FDR) or Gaussian random field (GRF) methods. For GICA analysis, group comparisons of component spatial Z-scores using two-sample *t*-tests with family-wise error (FWE) correction based on permutation testing. All *P*-values reported are two-tailed, and *P* < 0.05 was considered statistically significant.

## 3 Results

The study enrolled 68 patients with DLB and 38 age- and education-matched healthy controls. The demographic and cognitive assessment are summarized in Table 1, which shows that DLB patients and healthy controls were well-matched for demographic variables, with no significant differences in age at assessment, sex, education level, marriage, smoking, alcohol consumption, diabetes mellitus, hypertension, heart disease, and asymptomatic brain infarcts between the two groups (*P* > 0.05). However, compared to the control group, the DLB group exhibited significantly lower total scores on the MoCA (18.26 ± 3.45 vs. 26.84 ± 2.57, *P* < 0.001) and MMSE (21.37 ± 4.18 vs. 27.93 ± 1.96, *P* < 0.001).

**TABLE 1:** Demographic and cognitive assessment between the two groups.

Characteristics	DLB (*n* = 68)	NC (*n* = 38)	*P-*value
Sex (male *n*,%)	37 (54.4%)	18 (55.3%)	0.427
Age at assessment (years)	67.29 ± 7.48	67.17 ± 8.97	0.926
Education (years)	9.96 ± 4.35	9.83 ± 4.30	0.818
Marriage (*n*,%)			0.861
Married	56 (82.4%)	31 (81.6%)	
Divorced and widow	12 (17.6%)	7 (18.4%)	
Smoking, yes (*n*,%)	21 (30.9%)	14 (36.8%)	0.562
Alcohol consumption, yes (*n*,%)	24 (35.3%)	13 (34.2%)	0.894
Diabetes mellitus, yes (*n*,%)	16 (23.5%)	9 (23.7%)	0.898
Hypertension, yes (*n*,%)	19 (42.6%)	13 (34.2%)	0.205
Heart disease, yes (*n*,%)	8 (11.8%)	4 (10.5%)	0.872
Asymptomatic brain infarcts, yes (*n*,%)	11 (16.2%)	6 (15.8%)	0.831
MMSE	21.37 ± 4.18	27.93 ± 1.96	< 0.001
MoCA	18.26 ± 3.45	26.84 ± 2.57	< 0.001

DLB, Dementia with Lewy bodies; MMSE, Mini-Mental State Examination; MoCA, Montreal Cognitive Assessment; *P* < 0.05 significant difference.

**FIGURE 1:**
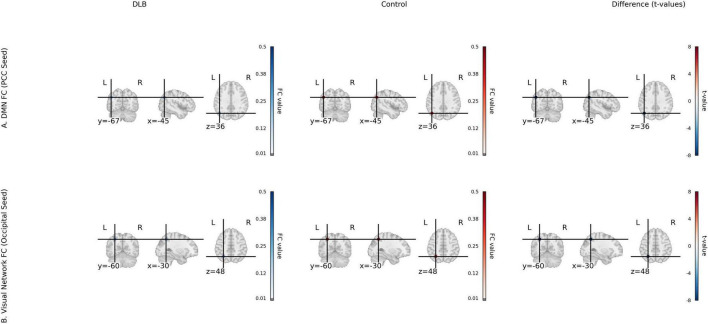
Functional connectivity (fcMRI) for DLB and control groups. **(A)** Default mode network (DMN) FC with PCC seed, showing group mean FC maps for DLB and Control, and a *t*-value map of group differences (t-statistics from two-sample *t*-tests) for PCC-AG and PCC-mPFC connectivity. **(B)** Visual network FC with occipital seed, showing group mean FC maps and *t*-value map for occipital-parietal connectivity. Color scale: *t*-values from –6 to 6 (magma colormap).

**FIGURE 2:**
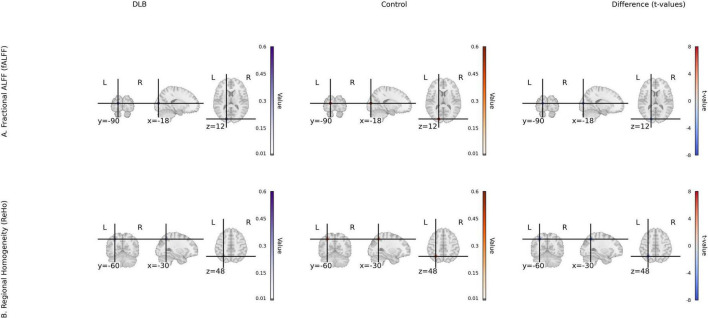
Local activity metrics for DLB and control groups. **(A)** Fractional ALFF (fALFF) group mean maps and *t*-value map of group differences in occipital, angular gyrus, and temporal pole. **(B)** Regional homogeneity (ReHo) group mean maps and *t*-value map of group differences in parietal and cerebellum. Color scale: *t*-values from –6 to 6 (magma colormap).

Seed-based functional connectivity analysis revealed significant reductions in the integrity of DMN in the DLB group compared to healthy controls. Specifically, functional connectivity was markedly decreased between the posterior cingulate cortex and the angular gyrus (PCC–AG: 0.25 ± 0.06 vs. 0.41 ± 0.07, *P* < 0.001) and between the PCC and the medial prefrontal cortex (PCC–mPFC: 0.27 ± 0.08 vs. 0.44 ± 0.06, *P* < 0.001), representing reductions of 38.19 and 38.64%, respectively (Table 2; Figure 1). In parallel, analyses of local neural activity showed significantly lower fALFF in DLB patients within key regions, including the posterior occipital lobe (*P* = 0.004), angular gyrus (*P* = 0.001), and left temporal pole (*P* < 0.001). ReHo was also significantly reduced in the left parietal lobe and posterior cerebellar lobe (both *P* < 0.001) (Table 2; Figure 2). These findings collectively indicate widespread impairments in both long-range network synchronization and local neural activity in DLB patients.

**TABLE 2:** Comparison of functional connectivity and local neural activity metrics between DLB and control groups.

Metric	DLB (*n* = 68)	NC (*n* = 38)	*P*-value
PCC–AG connectivity strength	0.25 ± 0.06	0.41 ± 0.07	<0.001
PCC–mPFC connectivity strength	0.27 ± 0.08	0.44 ± 0.06	<0.001
Left parietal ReHo value	0.31 ± 0.05	0.38 ± 0.06	<0.001
Posterior cerebellar ReHo value	0.29 ± 0.04	0.36 ± 0.06	<0.001
Posterior occipital fALFF value	0.39 ± 0.09	0.48 ± 0.08	0.004
Angular gyrus fALFF value	0.44 ± 0.08	0.51 ± 0.07	0.001
Left temporal pole fALFF value	0.37 ± 0.06	0.43 ± 0.06	<0.001

DLB, Dementia with Lewy bodies; PCC, Posterior Cingulate Cortex; AG, Angular Gyrus; mPFC, medial Prefrontal Cortex; ReHo, Regional Homogeneity; fALFF, Fractional Amplitude of Low-frequency Fluctuations; *P* < 0.05 significant difference.

**FIGURE 3:**
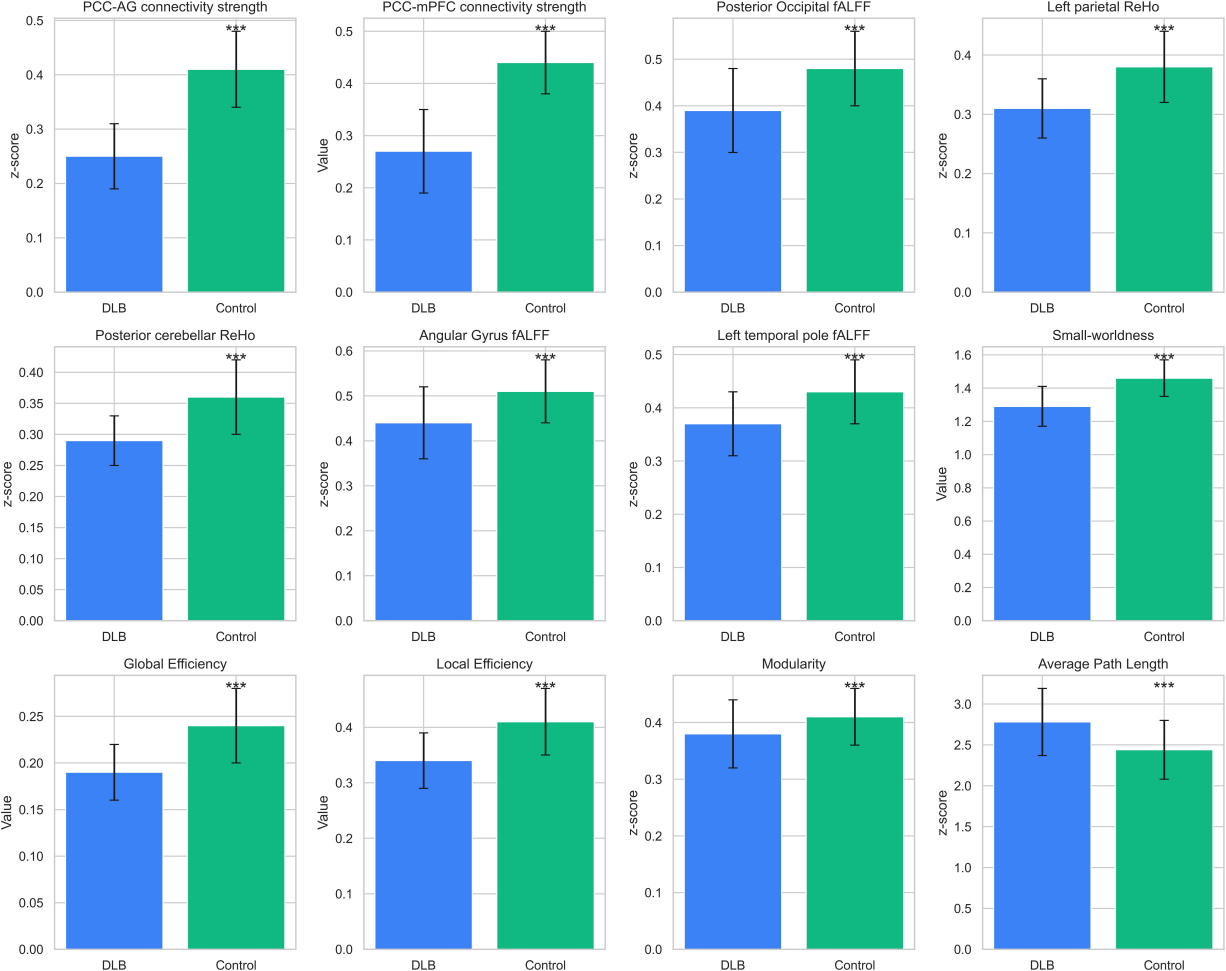
Functional neuroimaging differences between groups. Comparisons of functional neuroimaging metrics between DLB patients (*n* = 68) and healthy controls (*n* = 38). Metrics include functional connectivity (PCC–AG, PCC–mPFC), fALFF (posterior occipital, parietal), ReHo (parietal, cerebellar), and graph-theoretical measures (small-worldness, global efficiency). Bars represent mean ± SD, with significant differences (*P* < 0.001) marked by ***.

Graph theory analysis demonstrated significant disruptions in the global architecture of functional brain networks in DLB (Table 3; Figure 3). Compared to controls, the DLB group exhibited a significantly lower small-worldness coefficient (1.29 ± 0.12 vs. 1.46 ± 0.11, *P* < 0.001), global efficiency (0.19 ± 0.03 vs. 0.24 ± 0.04, *P* < 0.001), and local efficiency (0.34 ± 0.05 vs. 0.41 ± 0.06, *P* < 0.001). Conversely, the average path length was significantly prolonged (2.44 ± 0.36 vs. 2.78 ± 0.41, *P* < 0.001). These results indicate a shift toward a less integrated and less efficient functional network organization in DLB patients.

**TABLE 3:** Comparison of graph theoretical network metrics between DLB and control groups.

Metric	DLB (*n* = 68)	NC (*n* = 38)	*P*-value
Small-worldness coefficient	1.29 ± 0.12	1.46 ± 0.11	<0.001
Global efficiency	0.19 ± 0.03	0.24 ± 0.04	<0.001
Local efficiency	0.34 ± 0.05	0.41 ± 0.06	<0.001
Average path length	2.78 ± 0.41	2.44 ± 0.36	<0.001

DLB, Dementia with Lewy bodies; NC, normal control; *P* < 0.05 significant difference.

**FIGURE 4:**
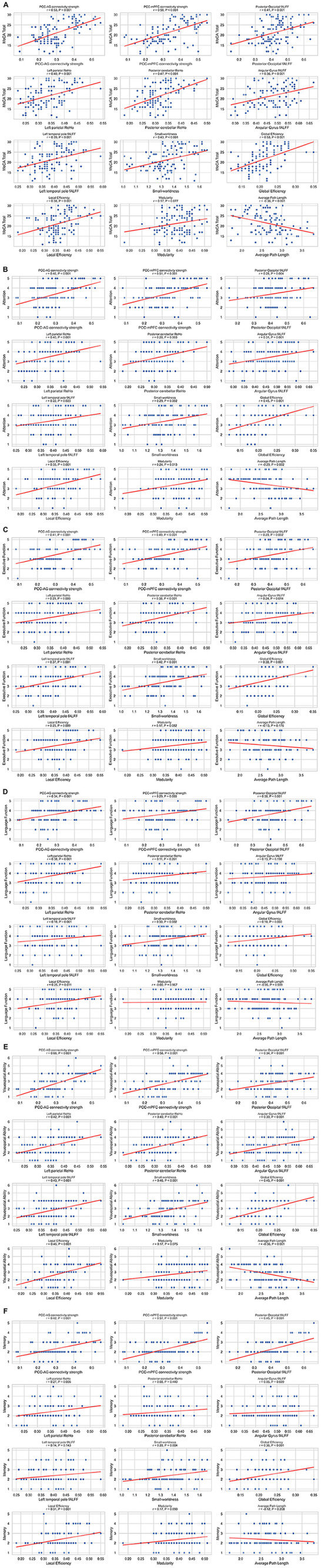
(**A–F)** Correlations between imaging metrics and clinical scales. Scatterplot matrix showing Spearman correlations between imaging metrics and clinical scales (MoCA total score, attention, executive function, language function, visuospatial ability and memory), *n* = 106. Each plot includes regression lines and correlation coefficients. Significant correlations (*P* < 0.05) are indicated.

Spearman correlation analyses revealed robust associations between key neuroimaging markers and cognitive deficits (Figures 4A–F). PCC–AG connectivity strength exhibited moderate-to-strong positive correlations with the MoCA total score (*r* = 0.53, *P* < 0.001), attention (*r* = 0.46, *P* < 0.001), executive function (*r* = 0.41, *P* < 0.001), and language function (*r* = 0.34, *P* < 0.001). Furthermore, posterior occipital fALFF and left parietal ReHo were significantly correlated with visuospatial ability (*r* = 0.34, *P* < 0.001 for fALFF; *r* = 0.42, *P* < 0.001 for ReHo) and memory function (*r* = 0.45, *P* < 0.001 for fALFF; *r* = 0.27, *P* = 0.006 for ReHo).

As shown in Table 4, multiple linear regression analysis with the MoCA score as the dependent variable identified PCC–AG connectivity strength (β = 0.58, *P* = 0.001) as the strongest predictor. The fALFF of the posterior occipital lobe (β = 0.31, *P* = 0.020), small-worldness coefficient (β = 0.27, *P* = 0.031), and education level (β = 0.22, *P* = 0.037) also emerged as significant predictors. The model explained 42% of the variance in MoCA total scores (*R*^2^ = 0.42, *P* < 0.001), with no severe multicollinearity detected [variance inflation factor (VIF) < 1.2 for all variables].

**TABLE 4:** Multiple linear regression analysis of MoCA scores.

Predator	β	SE	t	*P*	VIF
PCC–AG connectivity strength	0.58	0.17	3.37	0.001	1.21
Posterior occipital fALFF value	0.31	0.13	2.41	0.020	1.14
Small-worldness coefficient	0.27	0.12	2.22	0.031	1.19
Education level	0.22	0.10	2.14	0.037	1.05
Age	–0.15	0.08	–1.87	0.066	1.07

PCC, Posterior Cingulate Cortex; AG, Angular Gyrus; fALFF, Fractional Amplitude of Low-frequency Fluctuations; *P* < 0.05 significant difference.

**FIGURE 5:**
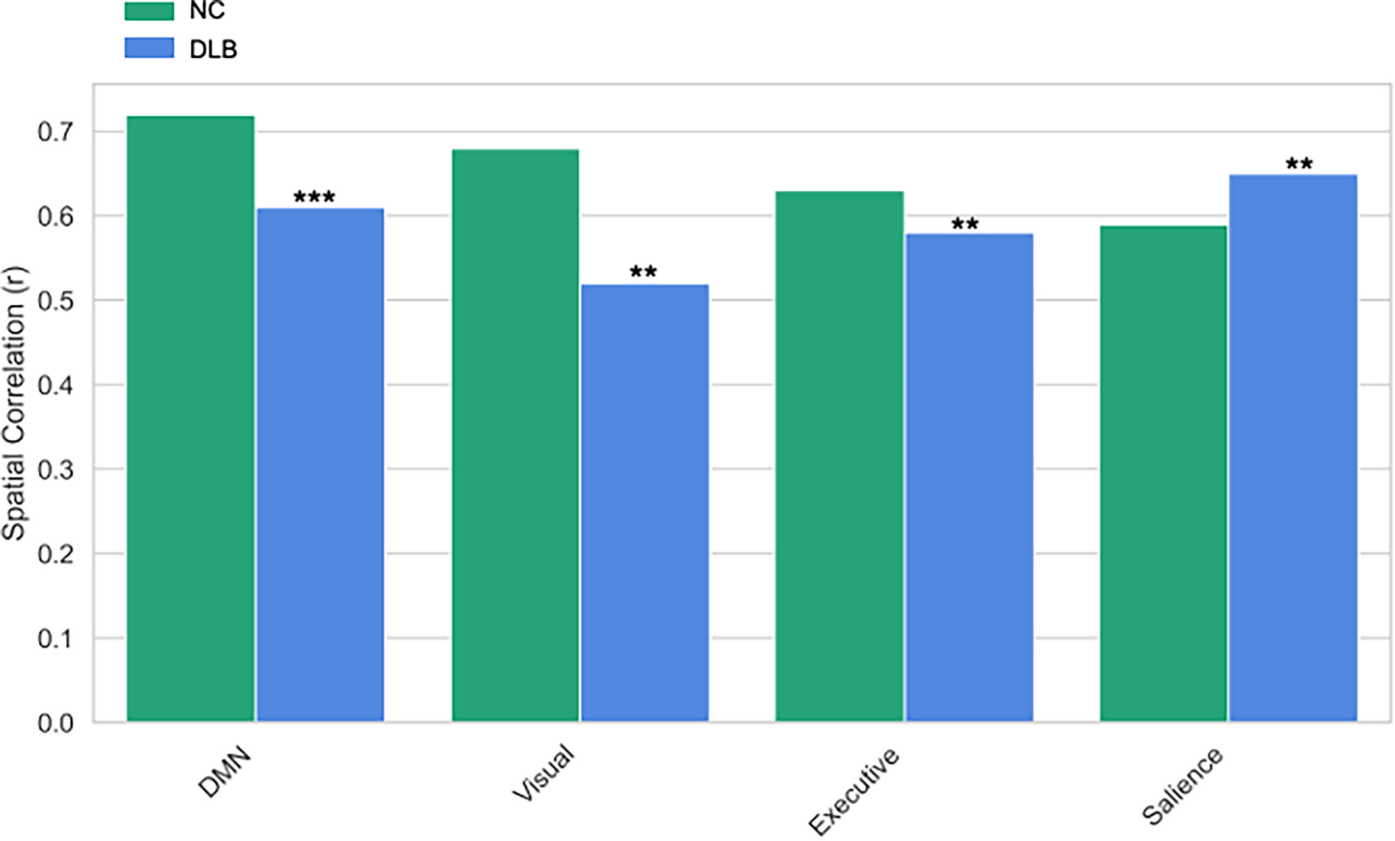
GICA network spatial correlation differences. The spatial correlation (Spatial Correlation, r) comparisons between the default mode network (DMN), visual network, executive network, and salience network in the Control (NC) group and the Lewy Body Dementia (DLB) group. *** and **indicates *P* < 0.001 and *P* < 0.01 respectively.

GICA identified 20 independent components, of which four core resting-state networks showed significant between-group differences (Figure 5). In the DMN, reduced activity in the posterior cingulate cortex/precuneus was shown in the DLB group [peak: (–5, –49, 40), *t* = 4.32, *P* < 0.001 FWE]. In the visual network, activity in the occipital pole (BA17/18) was decreased [peak: (–15, –93, 5), *t* = 3.87, *P* = 0.002 FWE]. In the executive control network, reduced dorsolateral prefrontal cortex activation was demonstrated in DLB [peak: (–46, 36, 28), *t* = 3.12, *P* = 0.008 FWE]. In contrast, the salience network exhibited enhanced activity in the anterior insula in DLB patients [peak: (34, 24, –2), *t* = 3.45, *P* = 0.003 FWE], potentially linked to attentional fluctuations. Spatial correlation analysis further confirmed reduced network specificity in the DLB group compared to controls, supporting these network-specific alterations.

## 4 Discussion

Our study demonstrates widespread FC disruptions in DLB, particularly within the DMN and visual network, characterized by pronounced decoupling between the PCC and key nodes such as PCC–AG and PCC–mPFC. These network-specific alterations not only distinguish DLB neuroimaging profiles but also mechanistically underpin its hallmark cognitive deficits, including fluctuating attention and visuospatial dysfunction.

The PCC–AG and PCC–mPFC, as core hubs of the DMN, play pivotal roles in maintaining self-awareness, episodic memory, and attentional modulation (Andrews-Hanna et al., 2010). Disruption of DMN connectivity likely impairs attention maintenance and information integration in DLB, contributing to clinical manifestations such as fluctuating attention and short-term memory deficits (Onofrj et al., 2019). Furthermore, diminished FC in the visual network may mechanistically explain the highly characteristic visual hallucinations in DLB. Supporting this, previous research (Lowther et al., 2014) reported reduced DMN activation and its correlation with cognitive fluctuations in DLB using rs-fMRI, while Kenny et al. (2012) identified PCC–AG connectivity loss as a key differentiator between DLB and Alzheimer’s disease (AD). Our findings extend prior work by quantifying the magnitude of connectivity decline, providing concrete parameters for DLB-specific neuroimaging biomarkers.

The integration of multimodal rs-fMRI metrics further revealed localized neural dysregulation. In this study, DLB patients exhibited significantly decreased fALFF and ReHo in the posterior occipital lobe, posterior cerebellum, and parietal regions, indicating both localized hypoactivity and desynchronized neural dynamics. Reduced fALFF and ReHo in posterior occipital and cerebellar regions imply diminished local neuronal synchronization and metabolic activity, potentially contributing to visuospatial deficits in DLB (Franciotti et al., 2015; Onofrj et al., 2019).

Our research findings in graph theory provide a system-level framework for understanding cognitive deficits in DLB. The reduction in small-world properties—evidenced by decreased global and local efficiency alongside increased path length—reflects a breakdown in the brain’s optimal network organization. This inefficient neural architecture impairs global information integration, contributing to deficits in attention and executive function, while also disrupting localized processing, thereby leading to impairments in memory and visuospatial abilities.

The graph-theoretical analysis further revealed compromised global network integration, characterized by reduced small-worldness, diminished global efficiency, and increased path length, collectively reflecting a systemic decline in functional network organization. Impaired whole-brain small-world properties—evidenced by decreased small-worldness coefficient, reduced global and local efficiency, and prolonged average path length—indicate a transition from an “efficiently integrated” network architecture to a “fragmented and inefficient” state, potentially driving multidimensional cognitive dysfunction. Some studies (Lowther et al., 2014; Sala et al., 2019) reported widespread functional connectivity reductions across resting-state networks in DLB, their studies did not systematically investigate topological properties. Complementarily, Chabran et al. (2020) identified co-occurring structural (gray matter atrophy) and functional connectivity abnormalities in DLB, suggesting structure-function covariation. Our findings extend these insights by employing a multidimensional analytical framework—integrating local activity metrics, functional connectivity, and graph theory—to validate a “network disintegration” phenotype in DLB, bridging microscale neuronal dysregulation and macroscale network collapse. By quantifying hierarchical topological degradation and linking it to clinical manifestations, this study provides a more granular understanding of DLB pathophysiology, offering a methodological advance over prior works focused solely on connectivity strength.

Notably, PCC–AG connectivity emerged as a robust independent predictor of cognitive impairment, correlating strongly with MoCA total scores and domain-specific deficits in attention and executive function. This pathway’s role in gating attention and integrating internal/external stimuli may explain DLB’s fluctuating cognition. Moreover, the synergistic predictive power of PCC–AG FC, occipital fALFF, and small-worldness highlights the complementary value of multimodal imaging in capturing DLB’s neurophysiological complexity (Zhou et al., 2008; Kucikova et al., 2024). While Babiloni et al. (2018) identified low-frequency EEG abnormalities in DMN and attention networks, and Chatzikonstantinou et al. (2021) linked FC deficits to attention deficits in DLB, our rs-fMRI-based multimodal analysis provides spatially precise, quantifiable biomarkers, a methodological advance with direct clinical applicability, to dissect cognitive predictors.

A vital next step is to determine whether the multimodal network disruptions we describe extend to the prodromal phase of DLB, such as in mild cognitive impairment with Lewy bodies or idiopathic REM sleep behavior disorder. Future research applying this integrated analytical approach to at-risk populations will be crucial to establish the predictive power of these biomarkers for conversion to overt dementia, a key translational objective for enabling early diagnosis and intervention.

This study has several limitations that should be acknowledged. First, the cross-sectional design precludes causal inferences about FC changes and disease progression, and the lack of longitudinal follow-up limits our ability to track how these network alterations evolve with clinical decline. Second, our findings are derived from a cohort of patients with established DLB, and they cannot speak to whether these specific multimodal fMRI alterations are present in the prodromal or preclinical stages of the disease. Third, the absence of AD or PDD control groups limits diagnostic specificity. Fourth, while our sample size (68 DLB patients) exceeds prior rs-fMRI studies, multicenter cohorts are needed to validate generalizability. Fifth, unaccounted confounders (e.g., neuropsychiatric comorbidities, medication effects) may also influence results. Finally, the use of MoCA and MMSE, while reliable for global cognitive assessment, limits the granularity of domain-specific cognitive evaluation. Future studies should integrate multicenter longitudinal designs to validate these biomarkers, explore FC-cognition causality, and develop predictive models for early DLB detection and personalized intervention.

## 5 Conclusion

This study integrated multimodal rs-fMRI metrics to reveal multilevel functional abnormalities in DLB, including disrupted connectivity in the DMN and visual network, altered local neural activity (e.g., posterior occipital and cerebellar regions), and impaired global network topology. Key imaging markers, such as PCC–AG connectivity strength, small-worldness coefficient, and posterior occipital fALFF, showed strong correlations with specific cognitive deficits, emerging as significant predictors of cognitive impairment in multivariate regression models. These metrics, characterized by high reproducibility and neurophysiological plausibility, establish a novel neuroimaging framework for DLB, offering actionable biomarkers to enhance early diagnosis and cognitive evaluation.

## Data Availability

The raw data supporting the conclusions of this article will be made available by the authors, without undue reservation.

## Footnotes

1
http://rfmri.org/dpabi

2
http://mialab.mrn.org/software/gift

3
https://www.nitrc.org/projects/gretna

## References

[B1] Andrews-Hanna J. R. Reidler J. S. Sepulcre J. Poulin R. Buckner R. L. (2010). Functional-anatomic fractionation of the brain’s default network. *Neuron* 65 550–562. 10.1016/j.neuron.2010.02.005 20188659 PMC2848443

[B2] Babiloni C. Del Percio C. Lizio R. Noce G. Lopez S. Soricelli A. (2018). Abnormalities of resting-state functional cortical connectivity in patients with dementia due to Alzheimer’s and Lewy body diseases: An EEG study. *Neurobiol. Aging* 65 18–40. 10.1016/j.neurobiolaging.2017.12.023 29407464

[B3] Bousiges O. Blanc F. (2022). Biomarkers of dementia with lewy bodies: Differential diagnostic with Alzheimer’s disease. *Int. J. Mol. Sci.* 23:6371. 10.3390/ijms23126371 35742814 PMC9223587

[B4] Caminiti S. P. Galli A. Jonghi-Lavarini L. Boccalini C. Nicastro N. Chiti A. (2024). Mapping brain metabolism, connectivity and neurotransmitters topography in early and late onset dementia with lewy bodies. *Parkinsonism Relat. Disord.* 122:106061. 10.1016/j.parkreldis.2024.106061 38430691

[B5] Chabran E. Noblet V. Loureiro, de Sousa P. Demuynck C. Philippi N. (2020). Changes in gray matter volume and functional connectivity in dementia with Lewy bodies compared to Alzheimer’s disease and normal aging: Implications for fluctuations. *Alzheimers Res. Ther.* 12:9. 10.1186/s13195-019-0575-z 31907068 PMC6945518

[B6] Chatzikonstantinou S. McKenna J. Karantali E. Petridis F. Kazis D. Mavroudis I. (2021). Electroencephalogram in dementia with Lewy bodies: A systematic review. *Aging Clin. Exp. Res.* 33 1197–1208. 10.1007/s40520-020-01576-2 32383032

[B7] Cieri F. Giriprakash P. P. Nandy R. Zhuang X. Doty R. L. Caldwell J. Z. K. (2024). Functional connectivity differences of the olfactory network in Parkinson’s disease, mild cognitive impairment and cognitively normal individuals: A resting-state fMRI study. *Neuroscience* 559 8–16. 10.1016/j.neuroscience.2024.08.031 39179019 PMC12975007

[B8] Folstein M. F. Folstein S. E. McHugh P. R. (1975). “Mini-mental state”. A practical method for grading the cognitive state of patients for the clinician. *J. Psychiatr. Res.* 12 189–198. 10.1016/0022-3956(75)90026-6 1202204

[B9] Franciotti R. Delli Pizzi S. Perfetti B. Tartaro A. Bonanni L. Thomas A. (2015). Default mode network links to visual hallucinations: A comparison between Parkinson’s disease and multiple system atrophy. *Mov. Disord.* 30 1237–1247. 10.1002/mds.26285 26094856

[B10] Fu Y. Halliday G. M. (2025). Dementia with Lewy bodies and Parkinson disease dementia - the same or different and is it important? *Nat. Rev. Neurol.* 21 394–403. 10.1038/s41582-025-01090-x 40355531

[B11] Geng L. Cao W. Zuo J. Yan H. Wan J. Sun Y. (2024). Functional activity, functional connectivity and complex network biomarkers of progressive hyposmia Parkinson’s disease with no cognitive impairment: Evidences from resting-state fMRI study. *Front. Aging Neurosci.* 16:1455020. 10.3389/fnagi.2024.1455020 39385833 PMC11461260

[B12] Grieder M. Wang D. J. J. Dierks T. Wahlund L. O. Jann K. (2018). Default mode network complexity and cognitive decline in mild Alzheimer’s disease. *Front. Neurosci.* 12:770. 10.3389/fnins.2018.00770 30405347 PMC6206840

[B13] Kenny E. R. Blamire A. M. Firbank M. J. O’Brien J. T. (2012). Functional connectivity in cortical regions in dementia with Lewy bodies and Alzheimer’s disease. *Brain* 135 569–581. 10.1093/brain/awr327 22189566 PMC3708629

[B14] Kucikova L. Kalabizadeh H. Motsi K. G. Rashid S. O’Brien J. T. Taylor J. P. (2024). A systematic literature review of fMRI and EEG resting-state functional connectivity in dementia with Lewy bodies: Underlying mechanisms, clinical manifestation, and methodological considerations. *Ageing Res. Rev.* 93:102159. 10.1016/j.arr.2023.102159 38056505

[B15] Lowther E. R. O’Brien J. T. Firbank M. J. Blamire A. M. (2014). Lewy body compared with Alzheimer dementia is associated with decreased functional connectivity in resting state networks. *Psychiatry Res.* 223 192–201. 10.1016/j.pscychresns.2014.06.004 25035300

[B16] Matar E. Ehgoetz Martens K. A. Phillips J. R. Wainstein G. Halliday G. M. Lewis S. J. G. (2022). Dynamic network impairments underlie cognitive fluctuations in Lewy body dementia. *NPJ Parkinsons Dis.* 8:16. 10.1038/s41531-022-00279-x 35177652 PMC8854384

[B17] McKeith I. G. Boeve B. F. Dickson D. W. Halliday G. Taylor J. P. Weintraub D. (2017). Diagnosis and management of dementia with Lewy bodies: Fourth consensus report of the DLB Consortium. *Neurology* 89 88–100. 10.1212/WNL.0000000000004058 28592453 PMC5496518

[B18] Nasreddine Z. S. Phillips N. A. Bédirian V. Charbonneau S. Whitehead V. Collin I. (2005). The montreal cognitive assessment, MoCA: A brief screening tool for mild cognitive impairment. *Am. Geriatr. Soc.* 53 695–699. 10.1111/j.1532-5415.2005.53221.x 15817019

[B19] Onofrj M. Espay A. J. Bonanni L. Delli Pizzi S. Sensi S. L. (2019). Hallucinations, somatic-functional disorders of PD-DLB as expressions of thalamic dysfunction. *Mov. Disord.* 34 1100–1111. 10.1002/mds.27781 31307115 PMC6707070

[B20] Peraza L. R. Kaiser M. Firbank M. Graziadio S. Bonanni L. Onofrj M. (2014). fMRI resting state networks and their association with cognitive fluctuations in dementia with Lewy bodies. *Neuroimage Clin.* 4 558–565. 10.1016/j.nicl.2014.03.013 24818081 PMC3984441

[B21] Piramide N. De Micco R. Siciliano M. Silvestro M. Tessitore A. (2024). Resting-state functional MRI approaches to Parkinsonisms and related dementia. *Curr. Neurol. Neurosci. Rep.* 24 461–477. 10.1007/s11910-024-01365-8 39046642 PMC11415422

[B22] Sala A. Caminiti S. P. Iaccarino L. Beretta L. Iannaccone S. Magnani G. (2019). Vulnerability of multiple large-scale brain networks in dementia with Lewy bodies. *Hum. Brain Mapp.* 40 4537–4550. 10.1002/hbm.24719 31322307 PMC6917031

[B23] Schumacher J. Peraza L. R. Firbank M. Thomas A. J. Kaiser M. Gallagher P. (2019). Dynamic functional connectivity changes in dementia with Lewy bodies and Alzheimer’s disease. *Neuroimage Clin.* 22:101812. 10.1016/j.nicl.2019.101812 30991620 PMC6462776

[B24] Vann Jones S. A. O’Brien J. T. (2014). The prevalence and incidence of dementia with Lewy bodies: A systematic review of population and clinical studies. *Psychol. Med.* 44 673–683. 10.1017/S0033291713000494 23521899

[B25] Walker Z. Possin K. L. Boeve B. F. Aarsland D. (2015). Lewy body dementias. *Lancet* 386 1683–1697. 10.1016/S0140-6736(15)00462-6 26595642 PMC5792067

[B26] Yamada Y. Kobayashi M. Shinkawa K. Nemoto M. Ota M. Nemoto K. (2022). Characteristics of drawing process differentiate Alzheimer’s disease and dementia with lewy bodies. *J. Alzheimers Dis.* 90 693–704. 10.3233/JAD-220546 36155515 PMC9697058

[B27] Yu E. Liao Z. Mao D. Zhang Q. Ji G. Li Y. (2017). Directed functional connectivity of posterior cingulate cortex and whole brain in Alzheimer’s disease and mild cognitive impairment. *Curr. Alzheimer Res.* 14 628–635. 10.2174/1567205013666161201201000 27915993

[B28] Zhou Y. (2019). *Multiparametric imaging in neurodegenerative disease.* Hauppauge, NY: Nova Science Publishers, 10.52305/UJVO6304

[B29] Zhou Y. Dougherty J. H. Hubner K. F. Bai B. Cannon R. L. Hutson R. K. (2008). Abnormal connectivity in the posterior cingulate and hippocampus in early Alzheimer’s disease and mild cognitive impairment. *Alzheimers Dement.* 4 265–270. 10.1016/j.jalz.2008.04.006 18631977

